# Effect of Different Cold Storage Temperatures on the Evolution of Shucking Yield and Quality Properties of Offshore Cultured Japanese Oyster (*Magallana gigas*) Treated by High Pressure Processing (HPP)

**DOI:** 10.3390/foods12061156

**Published:** 2023-03-09

**Authors:** Eduardo Puértolas, Sonia García-Muñoz, Mercedes Caro, Saioa Alvarez-Sabatel

**Affiliations:** AZTI, Food Research, Basque Research and Technology Alliance (BRTA), Parque Tecnológico de Bizkaia, Astondo Bidea, Edificio 609, 48160 Derio-Bizkaia, Spain

**Keywords:** high hydrostatic pressure, pressurization, bivalve mollusks, peeling, offshore farming, aquaculture

## Abstract

High pressure processing (HPP) can improve oyster shucking yield immediately after the treatment and increase the microbiological and sensory shelf life of oysters stored at 0–4 °C. However, the evolution of shucking yield during storage has not been previously examined and there are no studies focusing on shelf life at higher storage temperatures. To elucidate both aspects, control and HPP (300 MPa; 2 min) offshore cultivated oysters (*Magallana gigas*) were stored at 4 and 10 °C for 14 days, analyzing shucking yield, color, texture, microbiological and sensory characteristics. HPP samples showed a higher shucking yield (17% on average) than controls with minimal impact in texture and color, regardless of storage time and temperature. At 10 °C, HPP delayed microbial growth and sensory deterioration, increasing the estimated shelf life of oysters by 3 to 4 days (aerobic plate count < 6 log cycles; overall sensory acceptability > 2). Compared to controls stored at 4 °C, HPP oysters stored at 10 °C presented the same shelf life (5 to 9 days) but with higher shucking yield (up to 25%). In conclusion, HPP is an excellent tool to increase the shucking yield and delay sensory deterioration of oysters stored at 10 °C.

## 1. Introduction

Aquaculture is growing steadily, with mollusks being the third largest production category worldwide [1]. The most popular mollusk species by production is the oyster, which reached approximately 6.1 million tons in 2020, an increase of 44% compared to 2010 [1]. Among the different species, *Magallana gigas* (formerly *Cassostrea gigas*), also known as Pacific or Japanese oyster and produced in Asia, Europe, North America and Oceania, is one of the most consumed and appreciated. Oysters are usually farmed in coastal areas, such as in estuaries and inlets, frequently causing significant and unsolved problems such as pollution and coastal damage [2]. Oyster farming in the open sea (offshore production) is an emerging and promising approach that is gaining increasing attention to avoid these environmental problems, since the production takes place several kilometers from the coast rather than just a few meters away [2]. In addition, the excellent water quality of offshore systems is a major advantage over conventional farming in coastal areas, which is crucial for the performance and health of the farmed species [2]. Much literature has been published on the impact of offshore farming variables and physiological development of oysters [2,3,4,5]. However, there are no articles on the post-harvest sensory and microbiological quality of offshore oysters, let alone their evolution during storage, which may hinder the implementation of this farming system in the future.

High pressure processing (HPP) is an emerging technology that has gained interest in the seafood sector in the last two decades due to its broad range of applications, such as increasing shelf life of fresh and prepared meals, reducing allergenicity or reducing thawing losses [6,7,8,9,10]. It is an isostatic and adiabatic process that basically consists of pressurizing the food up to 800 MPa for a maximum of several minutes, keeping the temperature low throughout the process (typically < 25 °C), thus avoiding heat-mediated modification of food properties [11]. This non-thermal technology is used industrially for shucking oysters and other mollusks and crustaceans with minimal impact on sensory quality [11,12,13,14]. In oysters and other bivalve mollusks, HPP treatments denature their adductor muscle and, unable to contract, the shell opens spontaneously [11]. For this purpose, oyster processors typically use pressures between 200 and 350 MPa [11,12].

The application of HPP on oysters has been extensively studied by several researchers (Table 1), finding more advantages and possible uses on oysters than simple shucking. Thus, the use of HPP in raw oysters has been shown to be effective in reducing microbial load, including some pathogens such as *Vibrio* spp., increasing microbiological shelf life and reducing the risk from consumption of raw oyster [15,16,17,18,19,20]. Cruz-Romero et al. [20] observed that an HPP treatment of 260 MPa for 5 min reduced the total viable count of oysters by 0.9 log cycles, while pressures between 400 and 600 MPa lowered counts below the detection limit. Treatments at pressures of more than 300–350 MPa for 2 min can achieve more than 5 log reductions in *V. parahaemolyticus* [17]. Since the amounts of *V. parahaemolyticus* found in oysters are typically 3 log cycles, these treatments should be adequate to reduce the health risk of fresh consumption [17]. In addition to its antimicrobial effect, significant improvements in shucking yield have also been reported [12,20,21]. Cruz-Romero et al. [21] described that pressurizing oysters at 260 MPa for 3 min increased shucking yield by 15.5% over controls (shucking immediately after HPP treatment). However, neither these nor other authors have determined whether these improvements can be achieved in HPP oysters stored under refrigeration. This is of major interest since oysters for fresh consumption are usually stored and marketed whole and closed.

Oysters are a high-value and highly perishable product that is usually consumed raw and therefore must be stored at cold temperatures between 4 and 10 °C throughout the supply chain [19,22,23]. Despite the great potential of HPP to reduce microbiological risk and improve shucking yield and quality of oysters stored at relatively high temperatures such as 10 °C, there are surprisingly no published studies on this topic. In fact, most published studies used cold storage temperatures ranging from 2 to 5 °C [18,21,24,25,26,27,28] (Table 1). Furthermore, there is a lack of comprehensive studies comparing the quality evolution of fresh oysters treated with HPP and stored under refrigeration at different temperatures. In the only published study, oysters were stored at 5 °C or on ice, and only microbiological parameters were assessed [18].

**Table 1:** Summary of the main results of published papers on the use of HPP to improve oyster quality.

Reference	Key Parameters Investigated	HPP Treatment	Storage Conditions	Main Results
Koo et al. (2006) [16]	*V. parahaemolyticus*, *V. vulnificus*	207–345 MPa, 0–22 min	No storage	379 MPa, 6.5 min: >5 log reductions in *V. parahaemolyticus* 241 MPa, 5 min: >5.5 log reductions in *V. vulnificus*
Kural et al. (2008) [17]	*V. parahaemolyticus*	250 MPa, 5 min 300 MPa, 2 min 350 MPa, 1 min	No storage	>300–350 MPa, 2 min: >5 log reductions in *V. parahaemolyticus*
Ma et al. (2011) [18]	*V. parahaemolyticus,* aerobic and psychrotrophic plate count, coliforms	293 MPa, 1.5–3.5 min	5 °C; ice	293 MPa, 2 min: 3.52 log reductions in *V. parahaemolyticus*; shelf life of 6–8 days at 5 °C or 16–18 days in ice
Cruz-Romero et al. (2008) [20]	Aerobic and anaerobic plate count, H_2_S-producing bacteria, texture, color, lipid oxidation	260–600 MPa, 5 min	2 °C (ice)	HPP reduced microbial counts; increased lightness, cutting strength and lipid oxidation
Cruz-Romero et al. (2007) [21]	Proximate analysis, shucking yield, color	260 MPa, 3 min	No storage	260 MPa, 3 min: higher shucking yield; small differences in color; increased moisture; reduced protein
He et al. (2002) [24]	Aerobic plate count, sensory analysis	207–310 MPa, 0–2 min	4 °C	HPP: 2–3 log reductions in microbial counts; higher sensory scores during all storage
Liu et al. (2022) [25]	Fatty acid profile, total free amino acids, nucleotides, organic and inorganic ions	200–600 MPa, 3 min	4 °C; −20 °C	400–600 MPa, 3 min: no effect on fatty acid profile; decreased total free amino acids during storage; no changes in nucleotides, organic and inorganic ions
López-Caballero et al. (2000) [26]	Microbial flora (total viable count, H_2_S-producing bacteria, lactic acid bacteria, *Brochothrix thermosphacta*, coliforms), total volatile bases, texture	400 MPa, 10 min or 2 steps of 5 min	2 °C	400 MPa: up to 5 log reductions in microbial flora; increased total volatile bases; higher shear strength. HPP in 2 steps: no advantages
Rong et al. (2018) [27]	Sensory analysis, microbiological analysis (High-throughput screening), total volatile bases, lipid oxidation	300 MPa, 2 min	4 °C	300 MPa, 2 min: better sensory scores during storage; shelf life of 12 days (6–8 days for controls); increased lipid oxidation; *Psychrobacter* was dominant in the HPP oysters
Ye et al. (2015) [28]	Norovirus, sensory analysis, color, texture	300–600 MPa, 2 min	Ice	350 and 500 MPa, 2 min: >4 log reductions of GII.4 and GI.1 HuNoV; no changes in color and texture; higher overall sensory acceptability

Based on the above, the main objectives of the present study were (1) to determine whether the HPP-mediated improvements in shucking yield obtained following the treatments are maintained if the HPP oysters are stored under refrigeration and (2) to assess the impact of HPP on the shelf life of oysters stored under refrigeration at relatively high temperatures (10 °C). In addition, and importantly, this article aimed to study the evolution of the post-harvest sensory and microbiological quality of oysters cultivated in the open sea (offshore production). To achieve these objectives, the impact of HPP treatment on the quality evolution of offshore cultivated *M. gigas* oysters (shucking yield, color, texture, microbiological quality and sensory characteristics) stored at a relatively high cold temperature (10 °C) was analyzed. Control and HPP oysters were also stored at a relatively low temperature (4 °C) for comparative purposes. Considering that the use of HPP on offshore cultured oysters (*M. gigas*) has not been described previously, the HPP treatment was first optimized, guaranteeing a 100% opening rate with the best shucking yield and microbial load reduction, and with minimal impact on color, texture and sensory properties.

## 2. Materials and Methods

### 2.1. Schematic Overview of the Experimental Program

**Figure 1:**
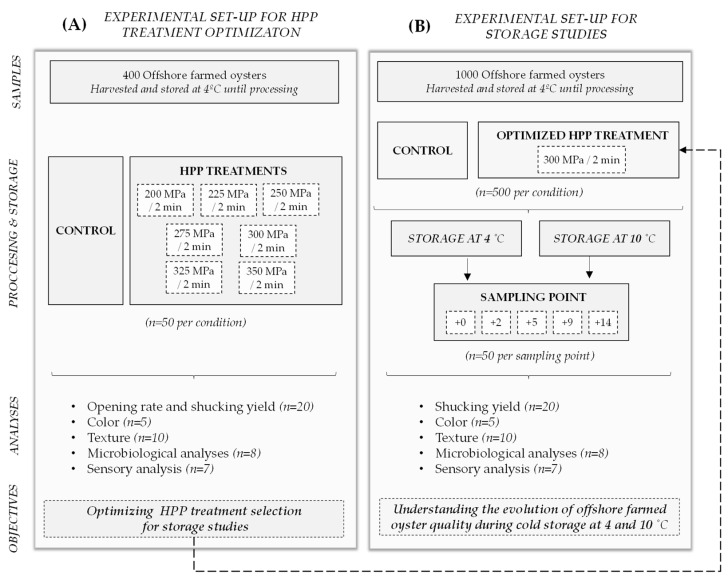
Schematic overview of the experimental program used in this work, for the optimization of the HPP treatment (**A**) and for the storage studies (**B**).

Figure 1 shows the experimental program used in this work. The optimal HPP treatment for processing oyster may vary with the species, the production conditions and the specific handling practices of each producer [11,21,24,27]. Since this is the first time that the application of this technology has been studied in oysters cultivated in the open sea (offshore production), a battery of tests was first performed to select the HPP treatment (Figure 1A). Once the HPP treatment was set, the evolution of oyster quality during cold storage at 4 and 10 °C was studied (Figure 1B).

### 2.2. Collection of Experimental Samples

Japanese oysters (*Magallana gigas*) were cultured in open waters (offshore farming) of the Basque coast (SE Bay of Biscay; 43°18′40.35′′, 2°22′36.03′′) [5] and harvested in October and November 2019, for HPP treatment selection and for storage studies, respectively. Oysters were transported to the AZTI pilot plant, stored at 4 °C and processed within 24 h of harvesting. Irrespective of the experiment, after reception, oysters were sorted in a cold room (4 °C) to obtain a uniform size. Each selected oyster was then brushed under running water to remove mud and other debris from the shell and was then tied with a rubber band to prevent the shells from opening during HPP treatments [28].

### 2.3. HPP Treatments

HPP treatments were performed in a 55 L high pressure unit (WAVE 6000/55HT; Hiperbaric, Burgos, Spain) previously described [6]. Water was used as pressurization medium. The inlet water temperature was 6 °C and chamber temperature was maintained below 18 °C during the whole process. HPP treatments were applied to the oysters at 200, 225, 250, 275, 300, 325 and 350 MPa for 2 min. Pressures were reached after 102, 111, 118, 132, 146, 153 and 162 s, respectively. In all cases the decompression time was less than 4 s. These HPP conditions were selected because, according to the literature, the optimal treatments for oyster processing are in this pressure range [17,18,21,24,27]. In fact, at the industrial level, pressures between 200 and 350 MPa and pressurization times between 2 and 3 min are used [11,12]. Higher pressures and processing times are not recommended as they cause excessive release of the adductor muscle in the shucking process [27].

For HPP treatment selection, 400 oysters (9.4 ± 1.3 cm (SD) shell length) were divided in 8 homogeneous batches of 50 individuals each, one for the control (without HPP treatment) and 7 for the different HPP conditions tested (200, 225, 250, 275, 300, 325 and 350 MPa for 2 min). After the HPP treatments, both control and HPP oysters were immediately analyzed (opening rate, shucking yield, color, texture, microbiological analysis, sensory analysis).

For the storage studies, oysters (1000 individuals; 9.7 ± 1.1 cm (SD) shell length) were homogeneously divided into 2 batches of 500 individuals, one for the control and one for the HPP (300 MPa for 2 min). After HPP treatment, the control and HPP batches were subdivided into 2 batches of 250 individuals each. One batch was stored at 4 °C and the other batch at 10 °C. After the different storage times (0, 2, 5, 9 and 14 days), for each storage temperature (4 and 10 °C) and processing conditions (control and HPP at 300 MPa for 2 min), 50 oysters were collected for analysis (opening rate, shucking yield, color, texture, microbiological analysis, sensory analysis). Storage temperatures (4 and 10 °C) were selected according to the minimum and maximum temperatures commonly found at the industrial level in the oysters’ supply chain [19,22,23].

### 2.4. Analytical Measurements

#### 2.4.1. Opening Rate and Shucking Yield

Opening rate and shucking yield were analyzed using 20 oysters of each condition studied. Each oyster was examined and opening effects were recorded as follows: no adductor muscle release (no opening), partial adductor muscle release (partial opening) and total adductor muscle release (full opening) [24]. Shucking yield was measured according to Cruz-Romero et al. [21]. After calculating the opening rate, oysters were shucked, drained in a stomacher filter bag (Seward, London, UK) and subsequently dried by placing the oyster tissue between 2 filter papers (Whatman Grade 1; Whatman International, Maidstone, UK) for 5 min. Shucking yield was expressed as mass of oyster tissue (g) per 100 g of whole closed oyster.

#### 2.4.2. Color

The lightness (L*), red-greenness (a*) and yellow-blueness (b*) of the ventral body surface of oysters [21,28] were determined using a colorimeter (Konica Minolta C-400, Tokyo, Japan) with a D65 illuminant and a standard observer angle of 10°. For each sampling condition, results were expressed as the mean of the measurements from 5 oysters.

#### 2.4.3. Texture

Oysters texture was determined instrumentally using an HD Plus texturometer (Stable Micro Systems, Surrey, UK). Due to the heterogeneity of oyster tissues, after some previous tests it was decided to measure texture in the gonadal region (ventral part of the oyster) using a 9 mm long Meullenet–Owens blade. The oyster body was placed in a plastic container and held so that it was immobilized during the measurements. Samples were cut with the blade specifically in the gonadal region, avoiding cutting other areas such as the mantle. Samples were cut to a depth of 10 mm at a speed of 10 mm/s.

All determinations were performed using a 5 kg load cell. For each type of treatment or sampling point, 10 samples were analyzed. The analyses allowed obtaining the force curves required to cut the samples, as well as the maximum cutting force (maximum force, N) and the work required to perform the cut (area under the curve, N·s). All the results of the texture determinations were analyzed with the Texture Exponent 32 software.

#### 2.4.4. Microbiological Analysis

For each sampling point, 10 g (and 25 g to detect *Salmonella* spp.) of oyster was homogenized with 90 mL of 0.1% buffered peptone water (Pronadisa, Laboratorios CONDA, Madrid, Spain) into sterile bags in a Stomacher 400 (Seward, UK) for three minutes. From the homogenate, samples were taken for the following procedures, preparing decimal dilutions series (when necessary) with buffered peptone water.

Aerobic (mesophilic) plate count (APC) was performed by pouring 1 mL of the corresponding dilution onto plate count agar (PCA, Pronadisa). After solidification of the agar, the plates were covered with 3–4 mL of melted PCA and incubated at 30 ± 1 °C for 48 h. For plate counts of *Enterobacteriaceae*, after homogenization, dilutions of oyster extracts (1 mL per plate) were assayed on poured plates of crystal-violet neutral-red bile glucose agar (VRBG, Oxoid, Hampshire, UK), which were prepared immediately prior to use, as specified by the manufacturer. After solidification of the agar, plates were covered with 3–4 mL of melted VRBG and incubated at 37 ± 1 °C for 24 h. After incubation, purple colonies were identified as *Enterobacteriaceae* and counted. For *Escherichia coli* (β-glucuronidasa positive), after homogenization, dilutions of oyster extracts (1 mL) were tested on 3M™ Petrifilm™ select *E. coli* count plate (SEC) (3M Microbiology Products, St. Paul, MN, USA). After incubation at 42 °C for 24 h, dark green to blue-green overgrown colonies were counted. The absence or presence of *Salmonella* spp. was tested by real time PCR (CFX96 Touch Real-Time PCR Detection System, Bio-rad Laboratories, Hercules, CA, USA) using the iQ-Check™ Salmonella II kit (Bio-rad Laboratories, Hercules, CA, USA).

*Vibrio* spp. counts were carried out based on Gopal et al. [29] by homogenizing 10 g of oyster meat in alkaline saline peptone water (Pronadisa) and seeding serial dilutions on thiosulfate-citrate-bile salts-sucrose agar (TCBS agar, Pronadisa). After incubation at 35 ± 1 °C for 48 h, the presence or absence of characteristic growth on TCBS agar was observed. Suspected *Vibrio* spp. should be either yellow colonies with turning of the medium to yellow due to sucrose fermentation, or green colonies, without medium turning for non-sucrose-fermenting species. Confirmation of *Vibrio* spp. was carried out by performing the Gram test, oxidase enzyme detection test, detection of indole production from tryptophan and sugar fermentation on triple sugar agar [29]. The calculation of the results was performed only considering the colonies with results compatible with the genus *Vibrio* in the characterization tests.

All microbiological counts were done in triplicate and expressed as log of colony forming units per gram of product (log CFU/g) except in the case of *Salmonella* spp., where the results were expressed as absence or presence of *Salmonella* spp. in 25 g.

#### 2.4.5. Sensory Analysis

Sensory quality was assessed by seven trained panelists [30] with extensive experience in seafood product evaluation, aged between 30 and 40, three women and four men. Trained panels of 5 to 9 members [13,24,27] are often used to assess complex product characteristics that are not possible to analyze with consumer panels. Products from the market were used to train the panelists. Filling degree, turgor, firmness, odor and overall acceptability were defined and evaluated. The filling degree was defined as the visual relation between the oyster tissue and the shell, from very high (the body of the oyster occupies most of the ventral surface of the shell) to very low (oyster body occupies approximately half of the ventral surface of the shell). Turgor was identified as the degree of swelling of the oyster tissue, from very swollen to flattened and wrinkled. The trained panel defined odor as that associated with the fresh oyster, from crisp hay to putrid odor. Firmness was defined as the amount of force required to deform the oyster with the index finger, from firm and elastic to squishy and deformable. The analysis was performed in individual sensory cabinets at the AZTI sensory lab [31]. The oysters (7 samples) were removed from cold storage 15 min before the test and left at room temperature. Samples were coded (randomized three-digit code) and served in random order. Assessment was performed on a 5 point scale from 1 to 5 (1: reject; 2: poor (sensory shelf life limit); 3: acceptable; 4: good; 5: excellent) [32].

### 2.5. Statistical Analysis

Results are expressed as mean values ±95% confidence interval (95% CI). The effect of different HPP treatments (control, 225, 250, 275, 300, 325 and 350 MPa) on oyster samples (*p* = 0.05) was analyzed by one-way ANOVA. The resulting *p*- and F-values are presented in the text where appropriate. The corresponding statistical differences between paired means were determined by Tukey’s test (*p* = 0.05). To analyze the effect of the optimal HPP treatment, storage time and storage temperature on the characteristics of oysters, the differences (*p* = 0.05) of paired means were obtained through Tukey’s test. Analyses were performed using Statgraphics Centurion software (Statpoint Technologies, Warrenton, VA, USA).

## 3. Results and Discussion

### 3.1. Selection of the HPP Treatment

Shucking is the main application of HPP in oyster processing [27]. The application is based on the denaturation of the adductor muscle, which results in the spontaneous opening of the shell [11]. The treatment exposes the meat, making it easier to remove, which results in a significant increase in yield and a better appearance of the oysters (no tissue remains attached to the shell). In addition, the risk of operator injury due to the conventional knife opening process is reduced [11,15]. If the HPP treatment is optimized, it allows not only shucking 100% of the oysters, but also reducing the natural microbial population, with minimal changes in their sensory quality [15,19]. This optimal HPP treatment depends, among others, on oyster species, growing conditions, oyster size and specific handling and processing practices [11,21,24,27]. Considering that the use of HPP on offshore farmed oysters has not been previously described, the HPP treatment was initially optimized to ensure a 100% opening rate with the best shucking yield, reducing microbial load but showing minimal impact on color, texture and sensory properties. For this purpose, pressures from 225 to 350 MPa were applied for 2 min to different batches of offshore farmed oysters (*M. gigas*) and compared to each other and to untreated (control) samples in terms of opening rate, shucking yield, color, texture, sensory characteristics and microbial quality (Table 2).

At 225 MPa, 30% of the oysters remained closed. Above 250 MPa, all oysters showed different opening levels and a pressure of at least 300 MPa was necessary to obtain 100% fully opened oysters. Rong et al. [27] found similar results in conventionally farmed *M. gigas* oysters, suggesting that oyster culture conditions (including farming method) may not be a really important factor in the shucking effect of HPP.

Oysters treated at 225, 250 and 275 MPa for 2 min presented similar shucking yields (g of oyster tissue/100 g of whole closed oyster) to the controls (*p* ≥ 0.05). However, the shucking yields showed by the oysters pressurized at 300, 325 and 350 MPa (15.02 ± 0.40, 14.44 ± 0.49, 15.32 ± 1.54 g/100 g, respectively) were significantly higher (*p* < 0.05) than those of control oysters (12.25 ± 0.49 g/100 g). These improvements (ranging from 17.8 to 25.1%) are within the levels reported in other studies with conventionally farmed oysters [12,20,21]. There are two main reasons: (a) increased water retention making the oyster more voluminous (increased moisture content) and (b) complete removal of the oyster from the shells (no loss of tissue) [15,20,21,26,28,33]. It is worth mentioning that the increase in moisture content is accompanied by a decrease in relative protein content (% wet weight) compared to raw oysters [21,34], so the consumer would be paying more for a product with higher weight, but with similar total protein content. The increase in moisture content only occurs when the oysters are in direct contact with water during HPP treatment. For example, He et al. [24] treated oysters in vacuum sealed waterproof bags and observed no water uptake in HPP oysters (207–311 MPa) compared to the control.

There was no significant difference (*p* ≥ 0.05) in L* (lightness) between control and HPP oysters at 225 MPa (Table 2). Pressures above 250 MPa resulted in an increase in L* compared to control samples (*p* < 0.05), with no differences observed among oysters treated in the 250–350 MPa range (*p* ≥ 0.05). This pressure-mediated change in L*, related to an increase in the opacity and whiteness, has been described by other authors for conventionally farmed oysters of different species [20,21,25,28]. It is essentially caused due to pigment alteration and protein denaturation/coagulation, which gives the product a cooked appearance [10,11,25]. In any case, the HPP treatments used for oyster shucking (<400 MPa) have less impact on color than heat treatments [21,28]. Although no differences (*p* ≥ 0.05) were detected in a* (red-greenness) among the control and HPP oyster groups (*p* = 0.99, F-value = 0.16), significant differences (*p* < 0.05) were detected in b* (yellow-blueness) (*p* = 0.01; F =3.87). If the results are studied in detail, the only significant difference detected was a higher b* in oysters treated at 350 MPa compared to the control and the rest of the HPP samples (*p* < 0.05).

Offshore farmed oysters tended to exhibit higher average cutting force and cutting work as the treatment pressure increased above 325 MPa (Table 2). However, although a trend was observed, no significant differences (*p* ≥ 0.05) were detected among the control and HPP oysters for either parameter (cutting force: *p* = 0.72, F-value = 0.61; cutting work: *p* = 0.33, F-value = 1.18). Most authors also reported non-significant differences in textural properties (just after treatment, not storage), between control and oysters treated at similar pressures [26,28,33]. These results were obtained irrespective of how and on which part of the oyster the texture measurements were made. For example, Yuyang et al. [33] did not observe differences in hardness (texture profile analysis; measurements made in the region of the adductor muscle) between control and treated oysters (*M. gigas*) at 100–300 MPa for 5 min. In contrast, other authors reported significantly higher cutting strength in oysters just treated with similar HPP conditions (260 MPa for 5 min) [20]. This behavior was attributed to protein aggregation induced by denaturation of myofibrillar proteins.

To determine the impact of HPP treatments on sensory properties, a trained panel of seven members was used. The application of pressures between 225 and 350 MPa did not influence the filling degree, firmness, odor and overall acceptance of the offshore farmed oysters (*p* ≥ 0.05) (Table 2). Before storage, similar results were also described by other researchers applying similar HPP treatments on conventionally farmed oysters [15,24,27]. Based on a trained panel of six panelists, Rong et al. [27] found no sensory differences in texture, fluid, color and odor between control and HPP oysters (*M. gigas*) at 100–350 MPa before storage. Although the oysters were not tasted by the panelists in this study, it is worth mentioning that, according to the literature, HPP oysters tend to retain the characteristic taste of raw oysters [12,15]. In some cases, even an enhancement of flavor has been described due to the uptake of salty water (increased water retention) [12,15].

It has been described that HPP can improve oyster shucking yield through increased water retention [21,22,28]. However, whether this is reflected in the sensory perception of oyster size/volume has not been measured before. Therefore, the impact of HPP treatments on turgor, defined by the trained panel as the degree of swelling of the oyster tissue, was analyzed. As a result, pressures higher than 300 MPa significantly increased the turgor value (*p* < 0.05) with respect to the control. This is consistent with the higher shucking yields found in oysters pressurized at 300–350 MPa (Table 2). It is noteworthy that despite these higher turgor values, oysters treated at these pressures (300–350 MPa) showed similar overall acceptability to the controls (*p* ≥ 0.05).

The aerobic plate count (APC) of control offshore farmed oysters was 3.88 ± 0.25 log cycles (Table 2). This value is slightly high but falls within the normal range published in the literature for fresh coastal farming oysters [18,24,26,27]. HPP is capable of inactivating vegetative microorganisms in food by denaturing proteins and enzymes, destabilizing cell walls and membranes and impacting DNA and ribosomal activity [10,11]. All HPP treatments tested (225–300 MPa for 2 min) significantly reduced APC by around 0.5–1 log cycle (*p* < 0.05), with values ranging from 2.84 to 3.07 log cycles. Similar reductions have been reported previously for comparable HPP treatments [18,24,27]. It is noteworthy that there were no statistical differences (*p* ≥ 0.05) for this parameter between the HPP groups of samples (*p* = 0.71, F-value = 0.59). This is because significantly higher reductions in microbial population usually occur at pressures higher than the maximum pressure applied in this work [11]. All other microbiological parameters examined (*Enterobacteriaceae*, *Vibrio* spp., *E. coli*, *Salmonella* spp.) were below detection limits for both the control and HPP samples, highlighting the good initial hygienic quality of the offshore farmed oysters.

Based on the results obtained, it is observed that HPP treatments ranging from 225 MPa to 275 MPa for 2 min reduced the APC of offshore farmed oysters (*M. gigas*) by about 0.5–1 log cycle compared to the controls, with minimal impact on color and without impact on texture and sensory quality. However, these treatments were not able to fully open the oysters and increase shucking yield. Pressures between 300 and 350 MPa were able not only to reduce the APC of offshore farmed oysters by about 0.5–1 log cycle, but also to obtain 100% fully opened oysters and a shucking yield between 17.8 and 25.1% higher than controls, with minimal impact on texture and sensory quality. Considering that there were no differences between offshore farmed oysters (*M. gigas*) treated between 300 and 350 MPa, the HPP treatment at 300 MPa for 2 min was selected for the storage studies (lower energy cost and shorter total processing time).

### 3.2. Effect of HPP and Cold Storage Temperature on Shucking Yield Evolution

Although several authors have described an increase in shucking yield (g of oyster tissue/100 g of whole closed oyster) due to HPP treatment [12,15,20,21], almost no studies have systematically explored the evolution of this parameter during cold storage. Kingsley [15] reported that the absorbed liquid, associated with increased shucking yield by HPP, did not remain within the bivalve tissues over time after shucking, but did not provide corroborating data, nor was it compared to a control.

**Figure 2:**
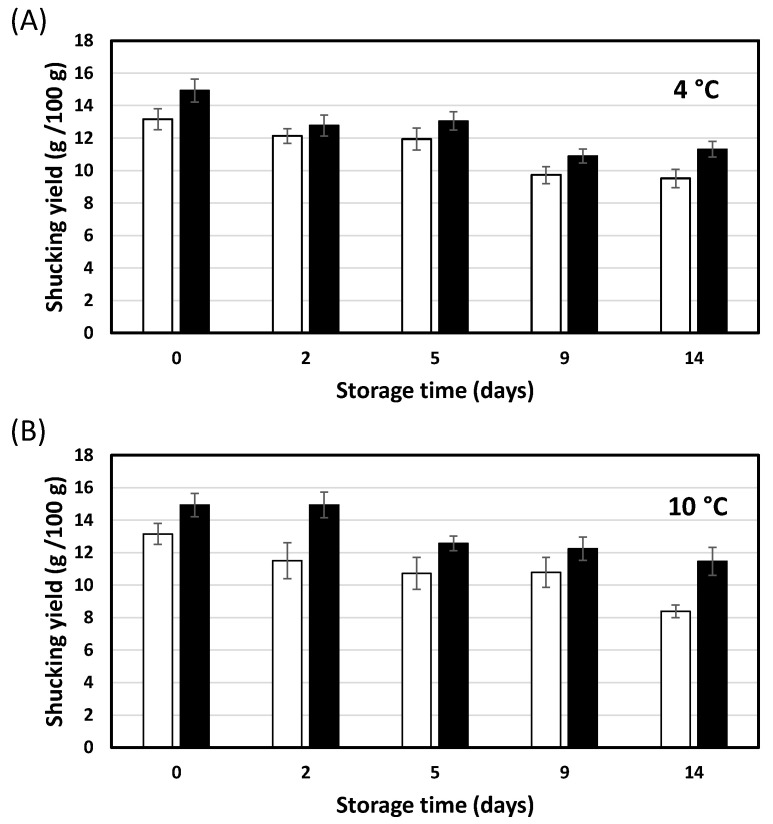
Shucking yield (g/100 g) of both control (white column) and HPP (black column) oysters (300 MPa for 2 min) during the 14 days of storage (sampling points: 0, 2, 5, 9 and 14 days) and at different storage temperatures (**A**) 4 °C and (**B**) 10 °C. All values are means ±95% CI (*n* = 20).

According to the results of the present work, the shucking yield of both control and HPP oysters (300 MPa for 2 min) progressively decreased during the 14 days of storage regardless of the storage temperature (4 or 10 °C) (Figure 2). For example, while the shucking yield of the offshore farmed oysters just after HPP was 14.92 ± 0.70 g/100 g, after 14 days of storage at 4 °C (Figure 2A) it was significantly reduced to 11.30 ± 0.49 g/100 g (*p* < 0.05). This decrease would be caused by a water loss during cold storage, related to the delicate characteristics of the oyster tissue. For example, within a few minutes of shucking oysters, oysters were observed to start losing juice [28]. He et al. [24] reported the absence of water loss (moisture content) in both control and HPP oysters (207–311 MPa) for up to 28 days of cold storage at 2–4 °C. However, these authors stored the oysters shucked instead of whole (closed with their shells), and packed in jars with water, so the difference in the procedure could be the cause of the difference in results.

Despite the progressive decrease in shucking yield during storage, a higher value (17% on average) was maintained in HPP samples compared to the controls, regardless of storage temperature and storage time. This improvement could be due to HPP-induced enhancement of protein hydration properties, which directly leads to increased water uptake during HPP treatment [14,33,34]. This change in protein would also allow HPP oysters to retain water better during storage compared to untreated oysters. Based on these improved hydration properties, it has been described that HPP can reduce the weight losses (increased yield) that occur during refrigerated and frozen storage of fish products [6,11,14]. Cartagena et al. [6] studied the application of HPP (200 MPa; 0–6 min) on albacore steaks prior to freezing to reduce weight loss during frozen storage. After 12 months of frozen storage, HPP albacore pretreated at 200 MPa for 6 min showed the same weight loss as the fresh control (2.46 ± 0.14 and 2.74 ± 0.30 g/100 g, respectively), while in the frozen control albacore it increased by 55.0% (4.25 ± 0.35 g/100 g). Further research is needed to elucidate the exact mechanism of water retention in pressurized seafood and to unlock its full potential to improve processing yields [14].

Storage temperature (4 and 10 °C) had no significant impact (*p* ≥ 0.05) on the shucking yield of both control and HPP samples, irrespective of storage time. Indeed, for both cold storage temperatures (4 and 10 °C), HPP allowed to reach a shucking yield after 14 days of storage (11.30 ± 0.49 and 11.46 ± 0.86 g/100 g, respectively) similar to the corresponding controls after 2 days of storage (12.13 ± 0.45 and 11.50 ± 1.10 g/100 g, respectively). This is of great interest, as it would indicate that the positive effect of HPP on shucking yield during 14 days storage would be independent of the cold storage temperature from 4 to 10 °C.

### 3.3. Effect of HPP and Cold Storage Temperature on Color Evolution

Samples processed by HPP (300 MPa for 2 min) were significantly lighter (increased L*) than control samples throughout the 14 days of storage, regardless of the cold storage temperature (4 or 10 °C) (*p* < 0.05) (Table 3). This is in agreement with the literature [21,25]. As explained above, the changes in pigments and proteins are mainly responsible for the increase in L* [10,11,25]. For both oysters stored at 4 and 10 °C, a* and b* values did not differ significantly (*p* ≥ 0.05) between control and HPP oysters (300 MPa) for each storage time (0, 2, 5, 9 and 14 days).

The L*, a* and b* values of the control and the HPP oysters (300 MPa) did not significantly change during the 14 days of storage, irrespective of the storage temperature (4 and 10 °C). The literature shows results very diverse in this respect. Cruz-Romero et al. [20] examined the changes in the color of oysters treated by HPP at 260, 400 or 600 MPa for 5 min and stored in ice (2 °C) for 31 days. These authors described increases during cold storage in the L* and b* values of control oysters (no differences in a*) while there were no significant changes in the color of HPP oysters, regardless of the pressure applied [20]. Applying similar HPP treatments (300, 450, 600 MPa; 3 min) and storage conditions (ice), other authors reported no or only small changes in oyster color parameters in both control and HPP oysters [28]. Liu et al. [25] applied HPP conditions comparable to those of this last work (200, 400 and 600 MPa; 3 min). They also described no significant differences in L* and b* values, but observed an increase in the a* value after 15 days at 4 °C in both control and HPP oysters (200, 400 and 600 MPa). The farming conditions, with special emphasis on feeding, could be one of the main reasons behind this diversity of results. Oysters, whether farmed conventionally (near the coast) or in the open sea (offshore production), feed naturally by filtering the water through their gills and can therefore develop different tones. Due to this natural feeding, there may even be differences between individuals of the same batch [28].

In conclusion, the color change (increase in L*) observed in offshore farmed oysters just after the HPP treatment (300 MPa) compared to the control was maintained for at least 14 days of storage at 4 or 10 °C. Furthermore, storage of oysters at 4 or 10 °C did not influence color (L*, a* and b*), with no significant differences (*p* ≥ 0.05) in equivalent samples in terms of treatment (control, HPP) and storage time (0, 2, 5, 9 and 14 days).

### 3.4. Effect of HPP and Cold Storage Temperature on Texture Evolution

Table 4 shows the evolution of cutting force and cutting work of the oyster tissue (gonadal region) during up to 14 days of storage at 4 and 10 °C for both control and HPP (300 MPa for 2 min) samples. It can be concluded that the storage temperature (4 and 10 °C), the storage time (up to 14 days) and HPP treatment (300 MPa for 2 min) did not affect the cutting force and cutting work of the offshore cultured oysters (*p* ≥ 0.05). These results are in agreement with those reported by Ye et al. [28], who found that control and pressurized (300–500 MPa for 2 min) oysters had similar textural characteristics (*p* ≥ 0.05) and these did not change during 15 days of ice storage (*p* ≥ 0.05). Other authors have reported a significant increase in shear strength with storage time for HPP samples compared to controls [20,26]. These authors applied HPP treatments of 5 [20] and 10 min [26], instead of the 2 min used in the present article and by Ye et al. [28]. The longer pressurization times applied by these authors [21,26] could be the reason for their different texture results. It is well known that the intensity of protein alterations caused by HPP, such as denaturation, aggregation and gelatinization, depends not only on the pressure applied but also on the pressurization time [11,14]. Therefore, if medium to high pressure (300–500 MPa) is applied, short pressurization times (e.g., 2 min) are necessary to achieve a texture similar to that of fresh oysters throughout refrigerated storage.

### 3.5. Effect of HPP and Cold Storage Temperature on Microbiological Quality Evolution

Oysters are bivalve mollusks that obtain the food they need by filtering water through their gills. In this process they may concentrate microorganisms [17]. Due to that, even with a careful handling and a proper storage, the shelf life of oysters is limited, typically around one week, causing practical problems for distribution and consumption [19,23,27]. Therefore, the potential of HPP to increase the shelf life of oysters, due to its ability to inactivate microorganisms at low temperatures, is of great interest to oyster producers. To examine the evolution of the microbial quality of control and HPP (300 MPa for 2 min) samples, aerobic plate count (APC), *Enterobacteriaceae*, *Vibrio* spp., *E. coli* and *Salmonella* spp. were determined over a maximum of 14 days of storage at 4 and 10 °C (Table 5). After 0, 2, 5 and 9 days of storage at 4 and 10 °C, HPP oysters had significantly lower APC (*p* < 0.05) than controls. The HPP treatment seems to delay the onset of the microbial growth phase in cold storage. Thus, the HPP treatment allowed maintaining the initial APC of offshore farmed oysters for 5 days at 4 °C (*p* ≥ 0.05) and for 2 days at 10 °C (*p* ≥ 0.05), while in the controls the APC values started to increase significantly after just 2 days of storage. Rong et al. [27] reported a similar delay (4 days) at 4 °C in conventionally farmed oysters treated with equivalent an HPP treatment (300 MPa; 2 min). HPP treatment also affected the growth of *Enterobacteriaceae*. While in controls stored at both 4 and 10 °C, *Enterobacteriaceae* counts above the detection limit (1 log cycle) were found just after 2 days of storage, in HPP samples it took 9 days at 4 °C and 5 days at 10 °C to observe this effect. This delay in microbial growth, and also a slow growth rate, have been observed in other seafood species [7,8,11,14], associated with sublethal damage to microbial cells, induced cellular stress and impact on DNA and ribosomal activity [10,26]. For example, a significant (*p* < 0.05) reduction in microbial growth (mesophilic bacterial counts, *Enterobacteriaceae*, psychotropic bacterial counts and *Pseudomonas* spp.) has been reported in salmon and plaice fillets treated at 500 MPa for 2 min [7]. The ability of the microorganisms to repair the damage would be the main factor that define the delay in microbial growth [10].

For both control and HPP oysters, *Vibrio* spp. and *E. coli* counts remained below the detection limit (1 log cycle) throughout the 14 days of storage, regardless of storage temperature. *Salmonella* spp. was not present at any combination of storage time and temperature. In any case, the ability of HPP to inactivate pathogens such as *Vibrio* spp., *E. coli* and *Salmonella* spp. and to prevent, or at least delay, the growth of these species during the shelf life has been described previously [16,18,25,26]. For example, HPP treatments ranging from 200 to 300 MPa can inactivate *Vibrio parahaemolyticus* and *Vibrio vulnificus* in oysters without compromising their sensory attributes [25]. On this basis, if a batch of freshly farmed oysters has high counts of these microorganisms, HPP could be a tool to reduce the initial microbial load, improving the quality of the oysters and allowing them to be sold. HPP is considered a non-thermal technology, as the temperature during the process is normally kept below room temperature [11]. To improve microbial inactivation, it is possible to apply HPP treatments at temperatures higher than 30–40 °C [11,15,17]. As a function of the temperature reached, this process is commonly referred to as pressure assisted thermal processing (PATP) or pressure assisted thermal sterilization (PATS) [11,13]. Processing at elevated temperatures damages the oysters, with changes in the quality being more than evident at temperatures higher than 50 °C [17]. Therefore, increasing the temperature of the HPP treatment would only be suitable for oysters if they are to be processed or sold cooked.

As long as pathogens are not present in sufficient doses in oysters, an APC limit between 5.70 and 6 log cycles has been suggested for freshly processed bivalve tissues to estimate their microbiological shelf life for human consumption [20,24]. Considering an APC limit of 6 log cycles, the estimated microbiological shelf life of the HPP oysters stored at 4 °C (9 to 14 days) would be longer than that of the control (5 to 9 days). As expected, the microbiological shelf life at 10 °C would be shorter than at 4 °C, although the estimated shelf life of HPP oysters (5 to 9 days) is still considerably longer than that of the controls (2 to 5 days). Looking at the data in detail, an HPP treatment of 300 MPa for 2 min would achieve at 10 °C the same estimated shelf life achieved by control oysters at 4 °C, between 5 and 9 days. This could be especially relevant in oyster supply chains where it is difficult to maintain low storage temperatures.

### 3.6. Effect of HPP and Cold Storage Temperature on Sensory Quality Evolution

**Figure 3:**
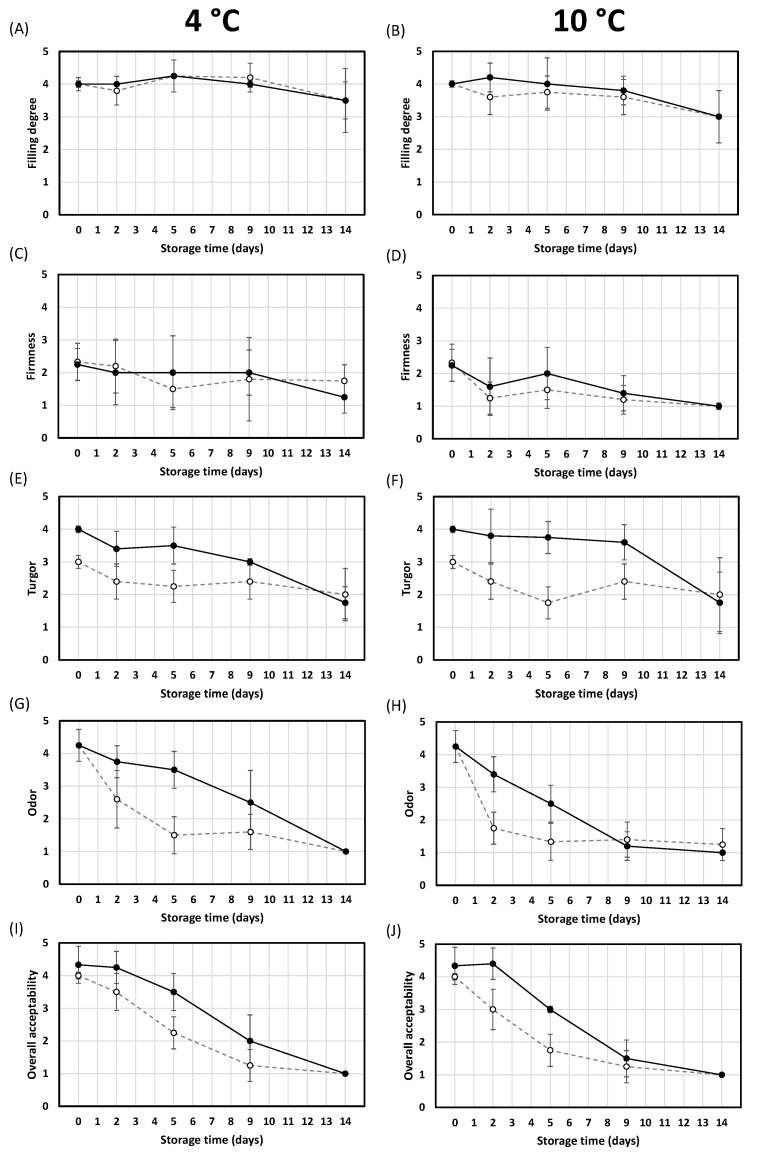
Evolution of the sensory quality (filling degree, firmness, turgor, odor and overall acceptability) during the storage at 4 °C (**A**, **C**, **E**, **G** and **I**, respectively) and 10 °C (**B**, **D**, **F**, **H** and **J**, respectively) of the oysters treated by HPP at 300 MPa for 2 min (closed symbols) and the respective controls (opened symbols). All values are means ± 95% CI (*n* = 7).

Figure 3 shows the evolution of sensory quality during storage of the oysters. Although the sensory quality was assessed by a trained panel, the confidence intervals obtained were relatively high. This has been also reported in previous works and has been related to the large differences between individual oysters, even within the same harvesting area and of similar shape and size [24].

The filling degree (Figure 3A,B) and firmness (Figure 3C,D) did not differ significantly (*p* ≥ 0.05) between control and HPP samples (300 MPa for 2 min), regardless of temperature (4 and 10 °C) and the storage time. Only one exception was observed for the firmness of control and HPP samples stored for 14 days at 10 °C, which decreased significantly (*p* < 0.05) compared to the initial samples (day 0). The results for firmness (no differences between control and HPP samples, irrespective of storage time and temperature) are in agreement with those obtained for the instrumental texture of the samples (Table 4). The absence of differences in the filling degree between control and HPP samples does not seem to correspond with the higher shucking yield of HPP oysters, irrespective of storage time and temperature (Figure 2). However, it should be noted that the filling degree was defined as the visual relation between the oyster tissue and the shell. In both control and HPP samples, the oyster body occupies a similar area of the ventral surface of the shell (values ranging from 3.5 and 4.3), so no differences in this parameter were detected.

Relevant differences in turgor, identified as the degree of swelling of the oyster tissue, were observed (Figure 3E,F). At both storage temperatures, HPP samples were perceived as more turgid than control samples (*p* < 0.05) up to 9 days of storage. As mentioned above, this sensory parameter would be directly related with the higher HPP-mediated water retention (which makes the oyster more voluminous) and, therefore, would be associated with the higher shucking yield of HPP oysters observed in this research (Figure 2). Regarding the influence of the storage temperature, no differences in turgor were detected between oysters stored at 4 or 10 °C for both control and HPP oysters.

Regardless of HPP treatment and storage temperature, odor scores decreased with storage time, with a more rapid decrease in samples stored at 10 °C (Figure 3H) compared to those stored at 4 °C (Figure 3G). While at the beginning of the storage study (0 days) all samples presented an excellent odor score (4.3), at 14 days all samples presented a putrid odor (value of 1). This behavior would be directly related to the high APC and *Enterobacteriaceae* counts detected in all samples at the end of storage (Table 5). Although at day 0 all samples obtained similar scores (*p* ≥ 0.05), HPP samples showed higher values between 2 and 9 days of storage at 4 °C and between 2 and 5 days at 10 °C (Figure 2G,H). As expected, this pattern reflects the evolution of APC and *Enterobacteriaceae* counts (Table 5).

At both storage temperatures, HPP oysters presented higher overall acceptability scores than control samples during 9 days of storage, although there were significant differences (*p* < 0.05) only during the first 5 days (Figure 3I,J). This would be mainly related to the higher odor and turgor scores of the HPP samples. Ye et al. [28] and Rong et al. [27] also described that HPP oysters have higher acceptability than control oysters during up to 8 days on ice or up to 14 days at 4 °C, respectively.

For estimating the sensory shelf life of oysters, the expert panel established a general acceptability limit value of 2 (poor). Taking this into account, the estimated sensory shelf life of the offshore farmed oysters stored at 4 °C would be 5 to 9 days for controls and 9 to 14 days for HPP samples. Rong et al. [27] obtained comparable results in conventionally farmed oysters treated and stored under similar experimental conditions (300 MPa for 2 min; storage at 4 °C). In this work, the control samples presented an unacceptable overall sensory score at day 8, while the score of HPP samples decreased slower and remained acceptable for up to 12 days [27]. Based on these results, and as expected, the sensory shelf life at 10 °C would be shorter than at 4 °C, although the estimated shelf life of HPP oysters (5 to 9 days) would still be considerably longer than that of the controls (2 to 5 days). Comparing all the data, it can be concluded that an HPP treatment of 300 MPa for 2 min would allow reaching at 10 °C the same estimated sensory shelf life (5 to 9 days) as that of the controls at 4 °C. These sensory shelf lives coincide with the microbiological ones estimated in this work and presented in the previous section.

## 4. Conclusions

The optimal HPP treatment for offshore farmed oysters (*M. gigas*) was 300 MPa for 2 min, resulting in a 100% opening rate, reducing APC by 1.07 log cycles while increasing shucking yield by 22.6% compared to untreated samples, with minimal impact on texture and overall sensory acceptability. As expected, the impact of HPP on offshore farmed oysters was similar to the impact of this technology on conventional farmed *M. gigas* oysters reported in the literature. This is noteworthy as no studies have been published to date on oysters produced using this culture technique.

For the first time, the effect of HPP treatment (300 MPa for 2 min) on the quality evolution of oysters (shucking yield, color, texture, microbiological and sensory characteristics) stored for 14 days at a relatively low temperature (4 °C) and at a high cold temperature (10 °C) has been compared. The HPP samples showed higher shucking yield (17% on average) than the controls during the 14 days of storage at both 4 and 10 °C, which has not been addressed before.

Considering microbiological and sensory quality limits (aerobic plate count < 6 log cycles; overall sensory acceptability > 2), the estimated shelf life of HPP oysters stored at 4 °C would be between 5 and 9 days for controls and between 9 and 14 days for HPP oysters. As expected, the shelf life at 10 °C would be shorter than at 4 °C due to the high rate of microbial growth and its impact on sensory quality (mainly odor), although the estimated shelf life of HPP oysters (5 to 9 days) would still be considerably longer than that of the controls (2 to 5 days). Based on these results, an HPP treatment of 300 MPa for 2 min would allow oysters to be stored at 10 °C, achieving the same microbiological and sensory shelf life (5 to 9 days) as controls stored at 4 °C, with minimal changes in texture and color, but maintaining a higher shucking yield (up to 25%). This could be relevant for oyster producers, especially when maintaining low storage temperatures (≤ 4 °C) throughout the supply chain is challenging.

Although the results are promising, HPP-mediated improvements in shucking yield during storage need to be verified in other species of oysters and bivalve mollusks. Oysters are sometimes not consumed fresh but are used as raw material in minimally processed or canned products. Therefore, the industrial use of HPP oysters should be studied to determine whether the improvements in shucking performance disappear or are maintained after processing. Finally, since HPP inactivates both pathogenic and spoilage microorganisms and delays microbial growth, this technology could minimize the microbiological risks of cold chain breakage in fresh oyster production. This hypothesis needs to be verified in future work.

## Figures and Tables

**Table 2:** Results obtained for untreated (control) and HPP oysters (225 to 350 MPa for 2 min) in terms of opening rate, shucking yield, color, texture, sensory characteristics and microbial quality.

	Control	225 MPa	250 MPa	275 MPa	300 MPa	325 MPa	350 MPa
Opening rate							
No open (%)	100	30	0	0	0	0	0
Partial open (%)	0	20	30	10	0	0	0
Full open (%)	0	50	70	90	100	100	100
Shucking yield (g/100 g)	12.25 ± 0.49 a	11.24 ± 1.89 a	12.54 ± 1.38 ac	12.11 ± 0.43 a	15.02 ± 0.40 b	14.44 ± 0.49 b	15.32 ± 1.54 bc
Color							
L*	51.48 ± 3.34 a	55.95 ± 3.81 ab	59.05 ± 2.39 b	57.77 ± 1.59 b	60.40 ± 3.64 b	59.23 ± 2.86 b	61.84 ± 3.58 b
a*	−1.24 ± 0.33 a	−1.31 ± 0.29 a	−1.20 ± 0.29 a	−1.21 ± 0.34 a	−1.23 ± 0.31 a	−1.37 ± 0.20 a	−1.29 ± 0.22 a
b*	10.68 ± 0.84 a	11.03 ± 0.98 a	10.78 ± 0.69 a	10.46 ± 0.51 a	11.25 ± 0.52 a	11.43 ± 0.58 a	12.69 ± 0.62 b
Texture							
Cutting force (N)	0.71 ± 0.26 a	0.50 ± 0.23 a	0.67 ± 0.24 a	0.71 ± 0.25 a	0.74 ± 0.26 a	1.25 ± 0.61 a	2.19 ± 1.63 a
Cutting work (N·s)	0.36 ± 0.11 a	0.25 ± 0.07 a	0.30 ± 0.08 a	0.33 ± 0.08 a	0.36 ± 0.06 a	0.56 ± 0.30 a	0.56 ± 0.31 a
Sensory analysis							
Filling degree	4.13 ± 0.26 a	4.00 ± 0.60 a	4.14 ± 0.51 a	4.00 ± 0.59 a	4.43 ± 0.58 a	4.29 ± 0.56 a	4.43 ± 0.58 a
Turgor	2.25 ± 0.34 a	2.86 ± 0.51 a	2.57 ± 0.40 a	2.67 ± 0.38 a	4.17 ± 0.56 b	4.29 ± 0.36 b	4.50 ± 0.41 b
Firmness	2.50 ± 0.79 a	2.86 ± 0.67 a	2.14 ± 0.67 a	2.00 ± 0.60 a	2.33 ± 1.01 a	1.71 ± 0.82 a	3.00 ± 1.13 a
Odor	4.13 ± 0.47 a	4.14 ± 0.51 a	4.00 ± 0.47 a	3.86 ± 0.67 a	3.71 ± 0.36 a	4.00 ± 0.60 a	4.17 ± 0.30 a
Overall acceptance	4.13 ± 0.47 a	4.00 ± 0.47 a	4.17 ± 0.56 a	3.83 ± 0.56 a	4.20 ± 0.62 a	4.00 ± 0.43 a	4.14 ± 0.51 a
Microbiology							
Aerobic plate count (log CFU/g)	3.88 ± 0.25 a	2.88 ± 0.25 b	3.07 ± 0.26 b	2.84 ± 0.26 b	3.03 ± 0.28 b	2.92 ± 0.26 b	3.07 ± 0.26 b
*Enterobacteriaceae* (log CFU/g)	<1	<1	<1	<1	<1	<1	<1
*Vibrio* spp. (log CFU/g)	<1	<1	<1	<1	<1	<1	<1
*Escherichia coli* (log CFU/g)	<1	<1	<1	<1	<1	<1	<1
*Salmonella* spp. (absence in 25 g)	-	-	-	-	-	-	-

All values are means ± 95% CI (opening rate and shucking yield *n* = 20; color *n* = 5; texture *n* = 10; sensory analysis *n* = 7; microbiological analysis *n* = 3). Different lowercase letters in the same row indicate significant differences (*p* < 0.05).

**Table 3:** L*, a* and b* values obtained for untreated (control) and HPP oysters (300 MPa for 2 min) over 14 days of storage (sampling points: 0, 2, 5, 9 and 14 days) and at different storage temperatures (4 and 10 °C).

	Storage Time (Days)	4 °C		10 °C	
		Control	300 MPa	Control	300 MPa
L*					
	0	53.22 ± 3.27 aA	61.62 ± 2.54 bAB	53.22 ± 3.27 aA	61.62 ± 2.54 bA
	2	50.99 ± 2.69 aA	63.44 ± 1.65 bA	53.05 ± 2.45 aA	61.67 ± 2.03 bA
	5	51.90 ± 2.23 aA	57.10 ± 2.22 bB	50.05 ± 2.98 aA	59.09 ± 2.90 bA
	9	51.50 ± 2.24 aA	61.65 ± 3.12 bAB	50.60 ± 2.59 aA	58.82 ± 1.81 bA
	14	54.01 ± 2.48 aA	60.36 ± 3.82 bAB	52.76 ± 2.66 aA	60.72 ± 2.31 bA
a*					
	0	−1.30 ± 0.51 aA	−1.27 ± 0.53 aA	−1.30 ± 0.55 aA	−1.27 ± 0.53 aA
	2	−1.20 ± 0.39 aA	−1.08 ± 0.64 aA	−1.33 ± 0.39 aA	−1.22 ± 0.66 aA
	5	−1.08 ± 0.34 aA	−1.16 ± 0.51 aA	−1.27 ± 0.32 aA	−1.17 ± 0.49 aA
	9	−1.31 ± 0.39 aA	−1.29 ± 0.39 aA	−1.38 ± 0.43 aA	−1.30 ± 0.36 aA
	14	−1.42 ± 0.32 aA	−0.92 ± 0.56 aA	−1.11 ± 0.56 aA	−1.35 ± 0.44 aA
b*					
	0	7.31 ± 1.61 aA	8.47 ± 1.42 aA	7.31 ± 1.61 aA	8.47 ± 1.42 aA
	2	6.94 ± 0.81 aA	8.13 ± 1.92 aA	6.46 ± 1.50 aA	8.55 ± 1.18 aA
	5	6.55 ± 1.40 aA	6.74 ± 2.04 aA	7.36 ± 1.80 aA	7.65 ± 1.82 aA
	9	6.91 ± 1.59 aA	6.53 ± 1.57 aA	7.60 ± 1.61 aA	6.77 ± 1.13 aA
	14	6.25 ± 1.28 aA	6.91 ± 0.76 aA	7.38 ± 0.55 aA	6.76 ± 0.75 aA

All values are means ± 95% CI (*n* = 5). Different lowercase letters in the same row indicate significant differences (*p* < 0.05). Different uppercase letters in the same column indicate significant differences (*p* < 0.05) for each color parameter in function of the storage time.

**Table 4:** Cutting force (N) and cutting work (N·s) values obtained from instrumental texture assessment for untreated (control) and HPP oysters (300 MPa for 2 min) over 14 days of storage (sampling points: 0, 2, 5, 9 and 14 days) and at different storage temperatures (4 and 10 °C).

	Storage Time (Days)	4 °C		10 °C	
		Control	300 MPa	Control	300 MPa
Cutting force (N)					
	0	0.72 ± 0.28 aA	0.60 ± 0.19 aA	0.72 ± 0.28 aA	0.60 ± 0.19 aA
	2	0.91 ± 0.26 aA	0.69 ± 0.21 aA	0.75 ± 0.28 aA	0.64 ± 0.11 aA
	5	0.67 ± 0.27 aA	0.61 ± 0.15 aA	0.76 ± 0.22 aA	0.60 ± 0.21 aA
	9	0.65 ± 0.25 aA	0.60 ± 0.29 aA	0.99 ± 0.50 aA	0.72 ± 0.24 aA
	14	0.62 ± 0.13 aA	0.69 ± 0.28 aA	0.83 ± 0.36 aA	0.83 ± 0.36 aA
Cutting work (N·s)					
	0	0.38 ± 0.19 aA	0.26 ± 0.05 aA	0.36 ± 0.19 aA	0.26 ± 0.05 aA
	2	0.39 ± 0.09 aA	0.35 ± 0.09 aA	0.36 ± 0.28 aA	0.26 ± 0.08 aA
	5	0.42 ± 0.18 aA	0.32 ± 0.06 aA	0.35 ± 0.06 aA	0.31 ± 0.10 aA
	9	0.30 ± 0.07 aA	0.28 ± 0.12 aA	0.46 ± 0.22 aA	0.33 ± 0.10 aA
	14	0.29 ± 0.05 aA	0.33 ± 0.12 aA	0.41 ± 0.13 aA	0.33 ± 0.09 aA

All values are means ± 95% CI (*n* = 10). Different lowercase letters in the same row indicate significant differences (*p* < 0.05). Different uppercase letters in the same column indicate significant differences (*p* < 0.05) for each texture parameter in function of the storage time.

**Table 5:** Results for the microbial quality (Aerobic Plate Count, *Enterobacteriaceae, Vibrio* spp., *Escherichia coli*, *Salmonella* spp.) obtained for untreated (control) and HPP oysters (300 MPa for 2 min) over 14 days of storage (sampling points: 0, 2, 5, 9 and 14 days) and at different storage temperatures (4 and 10 °C).

	Storage Time (Days)	4 °C		10 °C	
		Control	300 MPa	Control	300 MPa
*Aerobic Plate Count (APC)*					
	0	3.62 ± 0.33 aA	2.85 ± 0.23 bA	3.62 ± 0.34 aA	2.85 ± 0.23 bA
	2	4.22 ± 0.24 aB	2.93 ± 0.28 bA	5.87 ± 0.16 cB	3.04 ± 0.22 bA
	5	5.61 ± 0.35 aC	3.19 ± 0.43 bA	6.23 ± 0.20 cC	4.78 ± 0.23 dB
	9	6.46 ± 0.25 aD	5.36 ± 0.26 bB	7.04 ± 0.18 cD	6.38 ± 0.31 aC
	14	7.36 ± 0.19 aE	7.29 ± 0.28 aC	8.23 ± 0.36 bE	8.15 ± 0.21 bD
*Enterobacteriaceae*					
	0	<1	<1	<1	<1
	2	1.91 ± 0.35 aA	<1	1.60 ± 0.19 aA	<1
	5	2.60 ± 0.25 aB	<1	2.71 ± 0.30 aB	1.32 ± 0.22 bA
	9	3.20 ± 0.28 aC	2.55 ± 0.28 bA	4.52 ± 0.41 cC	3.96 ± 0.30 cB
	14	4.02 ± 0.33 aD	4.32 ± 0.39 aB	5.81 ± 0.37 bD	6.20 ± 0.34 bC
*Vibrio* spp.					
	0	<1	<1	<1	<1
	2	<1	<1	<1	<1
	5	<1	<1	<1	<1
	9	<1	<1	<1	<1
	14	<1	<1	<1	<1
*Escherichia coli*					
	0	<1	<1	<1	<1
	2	<1	<1	<1	<1
	5	<1	<1	<1	<1
	9	<1	<1	<1	<1
	14	<1	<1	<1	<1
*Salmonella* spp.					
	0	-	-	-	-
	2	-	-	-	-
	5	-	-	-	-
	9	-	-	-	-
	14	-	-	-	-

All values are means ± 95% CI (*n* = 3). “<1” indicates that the counts were below the detection limit (1 log cycle) and “-“ indicates the absence of *Salmonella* spp. in 25 g. Different lowercase letters in the same row indicate significant differences (*p* < 0.05). Different uppercase letters in the same column indicate significant differences (*p* < 0.05) for each microbial count in function of the storage time.

## Data Availability

Data supporting this study are available on reasonable request from the corresponding author.
